# Simulated Microgravity: Critical Review on the Use of Random Positioning Machines for Mammalian Cell Culture

**DOI:** 10.1155/2015/971474

**Published:** 2015-01-14

**Authors:** Simon L. Wuest, Stéphane Richard, Sascha Kopp, Daniela Grimm, Marcel Egli

**Affiliations:** ^1^Lucerne University of Applied Sciences and Arts, School of Engineering and Architecture, CC Aerospace Biomedical Science and Technology, Space Biology Group, Lucerne University of Applied Sciences and Arts, Seestraße 41, 6052 Hergiswil, Switzerland; ^2^Institute of Biomedicine, Pharmacology, Aarhus University, Wilhelm Meyers Allé 4, 8000 Aarhus C, Denmark

## Abstract

Random Positioning Machines (RPMs) have been used since many years as a ground-based model to simulate microgravity. In this review we discuss several aspects of the RPM. Recent technological development has expanded the operative range of the RPM substantially. New possibilities of live cell imaging and partial gravity simulations, for example, are of particular interest. For obtaining valuable and reliable results from RPM experiments, the appropriate use of the RPM is of utmost importance. The simulation of microgravity requires that the RPM's rotation is faster than the biological process under study, but not so fast that undesired side effects appear. It remains a legitimate question, however, whether the RPM can accurately and reliably simulate microgravity conditions comparable to real microgravity in space. We attempt to answer this question by mathematically analyzing the forces working on the samples while they are mounted on the operating RPM and by comparing data obtained under real microgravity in space and simulated microgravity on the RPM. In conclusion and after taking the mentioned constraints into consideration, we are convinced that simulated microgravity experiments on the RPM are a valid alternative for conducting examinations on the influence of the force of gravity in a fast and straightforward approach.

## 1. Introduction

Gravity is an omnipresent force on Earth, and all living organisms have evolved under the influence of constant gravity. Some organisms have learned to take advantage of the force of gravity by using it as a reference for orientation. The condition of microgravity (or near weightlessness) and its effects on living organisms, on the other hand, have always presented a fascinating scenario in biology and medicine. With the first manned space flights, it became clear that the human organism reacts with a series of adaptations to microgravity. Interestingly, some of the symptoms observed in space, such as wasting muscle mass and decreasing bone density, are typically diagnosed in the elderly as well [1–3]. This is one important factor that fostered scientists' interest in doing space research.

Numerous studies on mammalian organisms, for example, have demonstrated that the absence of gravity has severe effects not only on a systemic level but also on a cellular level. Short-term effects of microgravity (on the order of seconds) can be studied on research platforms such as drop towers or airplanes that fly in parabolic maneuvers. In contrast, long-term effects can only be studied on board sounding rockets (on the order of minutes) and space vehicles in flight. Due to the extensive preparation effort, safety constraints and rare flight opportunities, however, access to space experiments is limited. For many years, the random positioning machine (RPM), besides other tools, has been successfully used to simulate microgravity for screening studies, pre- and postflight experiments, and hardware testing. The principle of the RPM (a specialized, two-axis form of the clinostat) is based on gravity vector averaging to zero [4]. The typical RPM system comprises two gimbal-mounted frames, which are each driven by independent motors. Through dedicated algorithms, the samples placed on the inner frame are constantly reoriented, such that the gravity vector is distributed in all directions over time. Thus, from the sample's point of view the constantly reorienting gravity vector's trajectory averaged over time shall converge toward zero. However, 1 g is always acting on the sample at any given instant. It is assumed that the gravity vector needs to point in a specific direction for a minimal period of time in order to allow biological systems, like cells, to adapt to the gravity vector. But if the gravity vector constantly changes its orientation, the cells will lose the sense of direction and thus experience a state similar to microgravity (removed gravity vector). Therefore, the rotation of the frames shall be faster than the biological process studied [5]. However, the rotation cannot be too fast, as centrifugal forces will become effective [6]. Therefore, the RPM is typically used to examine slow processes, which are observed at least on the timescale of hours. It remains a legitimate question whether the RPM can reliably simulate microgravity. In this review we attempt to provide an answer to that question by comparing data of mammalian cells obtained at real microgravity in space and at simulated microgravity generated by using the RPM. In the first part, however, a summary is provided on the latest technical development as well as new applications of the RPM.

## 2. RPM Development and Technology

### 2.1. RPM Systems

**Figure 1:**
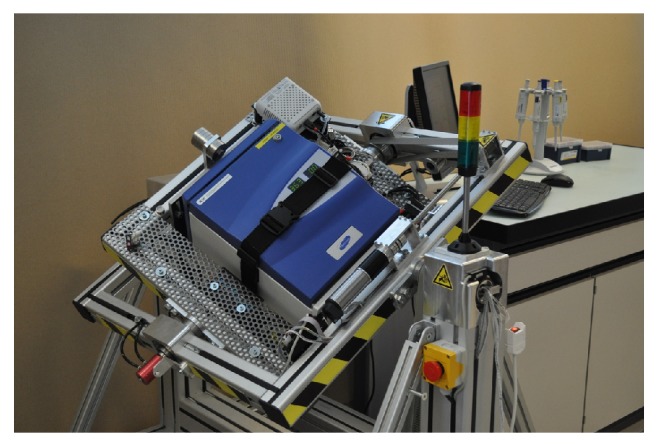
Random positioning incubator (RPI) featuring a fully integrated CO_2_ incubator (developed by the Institute for Automation, University of Applied Science Northwestern Switzerland).

Today's RPMs were introduced by Japanese plant researchers for conducting their particular studies [7, 8]. Later on, a similar machine was developed in The Netherlands (Dutch Space) [5]. Although both systems were commercialized [6], their range of use for doing space-related experiments was limited. For instance, scientific studies with mammalian cells that are very sensitive to temperature fluctuations were difficult to carry out because of a missing temperature control unit. Thus, these kinds of experiments had to be operated in a temperature-controlled room (e.g., a growth chamber). One approach to overcome this limitation was to miniaturize the RPM to fit into an ordinary cell culture incubator (max. size 50 × 50 × 50 cm) that offers precisely controlled temperatures (also referred to as desktop RPM) [4]. Through this RPM modification, the installation of large climate chambers around RPMs became unnecessary. We have recently reported another approach to upgrading the RPM by installing a commercial CO_2_ incubator onto the rotating frames. This RPM, called “random positioning incubator” (RPI) [9, 10], has the advantage of being independent of large laboratory incubators (Figure 1). Furthermore, the closed chamber of the incubator isolates the environment of the culture flasks and thus prevents exposure of biological samples to vapor and wear from the machinery, for example, [10]. Besides differences in the design of the three RPM types (regular RPM, small desktop RPM, and RPI), there appear to be slightly different concepts of how to average the gravity vector. The algorithm implemented on the Japanese RPM (referred to as a regular RPM) lets the RPM run with random rotational speeds and changes the velocity after two possible predefined periods (e.g., 30 or 60 s) [8]. The Dutch systems (referred to as regular and desktop RPMs) rotate with random speeds that are varied at random time points [6]. In contrast, our RPI rotates with constant velocity, but the rotation direction is inverted at random time points. The transition from forward to backward takes place at a predefined rotational acceleration [10]. All three algorithms employed by the different RPM types are reported to be reliable in averaging gravity. To our current knowledge, these algorithms are equivalent from a biological point of view.

### 2.2. Live Cell Imaging on the RPM

Microscopy is a common analytical tool used in cell biology. Even though microscopes are used on clinostats (rotation around one horizontal axis) [11, 12], until recently live cell imaging was not successful on an operating RPM. To date, most of the optical microscopy techniques applied under simulated microgravity conditions have been realized in the field of physical sciences. For such experiments, microscopes with a low numerical aperture and poor imaging performances were used because of their intrinsic robustness to environmental disturbances such as vibrations. In life science, however, high magnification is needed to detect modifications at the cellular or subcellular level. Because most of the ground-based microgravity research platforms are not vibration-free, high-performance microscopy has not been applicable. Thus, studies involving cell imaging have been conducted in ground laboratories after chemical fixation of the cell in microgravity. This approach implies a series of static shots, which cannot truly reveal the dynamic processes and labile cellular events occurring in cells in response to microgravity exposure.

Until recently, there was no system available that allowed high-quality real-time images taken at cellular or subcellular level under (real or simulated) microgravity. The breakthrough came with the use of a digital holographic microscope (DHM) that we have combined with an epifluorescent microscope. In this dual-mode microscope, the two imaging modes (DHM and fluorescent) operate sequentially. The DHM is an innovative interferometric microscope that is less sensitive to vibrations. The technological advantages of the DHM, which comprise continuous and fast digital autofocusing with a short exposure time, allow high-resolution imaging [13–16]. We tested the DHM on the RPM as well as during parabolic flights, and in both cases we obtained good data [13–16]. For instance, we followed reorganization of the actin cytoskeleton and fluctuations of the intracellular calcium concentration under simulated microgravity (unpublished data).

## 3. Partial Earth Gravity Load

During past years, RPM development was focused on the improvement of the hardware. We have also been working on an upgrade of the software responsible for controlling the motion of the RPM. Three different algorithms were introduced recently that simulated partial Earth gravity (0 to 0.6 g), allowing simulation of moon- or Mars-like gravity conditions [9]. All algorithms are adaptations of the random walk algorithm originally designed to simulate microgravity [10]. As described above, simulated microgravity is achieved by rotating both frames with constant velocity and inverting the rotation direction at random times. Partial gravity is achieved by altering the random walk in a way that the Earth's gravity vector is not completely randomized anymore and points (from the sample's point of view) for a prolonged time in a specific direction. In one algorithm version, this is accomplished by slowing down the rotational velocity while the gravity vector (considered in the sample frame) is pointing downwards. Otherwise, the frames rotate with the predefined velocity. The ratio of the two velocities finally determines the mean gravity (gravity vector averaged over time). In the other two algorithm versions, the random walk is interleaved with static intervals in which the frames stand still in a predefined orientation. However, the timing of these static intervals (start point and duration) is handled differently. In one case the timing is flexible and adjusted online as the experiment runs. In the other case the timing is strictly periodic and predefined before the experiment starts [9].

All three algorithms were tested on suspended human T cells and adherent mice myoblasts. Chemically activated T cells showed a decreased activation rate that correlates strongly to the decreasing simulated mean gravity values [9]. The results were similar for all algorithms. The adhered myoblast (C2C12 cell line) showed a decreased proliferation rate with decreasing mean gravity [9]. Interestingly, this effect is algorithm dependent. The correlation between mean gravity and proliferation was reduced or disappeared in the two algorithms involving static intervals [9]. Ideally, these types of partial gravity experiments are carried out in space by using a centrifuge. To our knowledge, no comparable space experiments have been conducted so far, except during particular parabolic flight campaigns of the European Space Agency (ESA). Therefore, a direct comparison to space is not possible at this time. However, these experiments demonstrate that simulation of partial gravity opens a new field of scientific questions that attracts other research groups. Dutch Space was attracted by the new topic as well and thus recently introduced a modified desktop RPM (presented at the ELGRA meeting 2013) allowing partial gravity simulations. Partial Earth gravity enabling RPMs increase the application range substantially, allowing investigation of the influence of gravity—like on the moon or Mars, for example,—on cells and small organisms at affordable cost. These results may help to estimate the biological response of cells or even whole organisms when exposed to the gravity loads of other planets or moons.

## 4. RPM Use and Experiment Quality

### 4.1. Cultivation Method of Mammalian Cells

**Figure 2:**
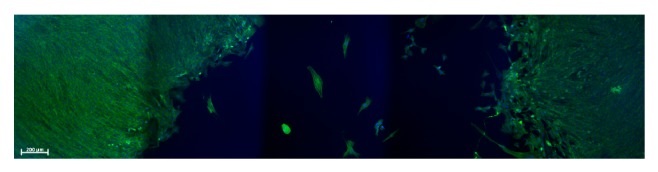
Mouse myoblasts (C2C12 cell line) were cultured until near confluence and subsequently exposed to a frequently passing air bubble. The culture chamber filled with medium was swinging upside down, such that the intentional air bubble frequently passed the same trajectory. The sample was fixed and stained for actin (green) and DNA (blue) thereafter. The cells in the trajectory of the air bubble got detached from the substrate (dark central area), while cells in the unaffected area kept proliferating (lateral green areas). Interestingly, detached cells could reattach to the opposite side of the culture chamber. Measuring bar 200 *μ*m. (Due to the limited field of view, this image has been stitched together from five images.)

In order to obtain comparable data, it is important to standardize cell culture methods. One of the most important aspects of doing so is a stable cultivating environment. When cultivating cells on the moving RPM, additional aspects have to be considered, such as avoiding air bubbles in the culture chambers [4]. Experiments have shown that an air bubble passing by adherent cells at the same trajectory repetitively (as the culture chamber moves in a “swinging motion”), the cells can detach from the substrate (Figure 2, unpublished observation). Interestingly, these cells often reattach at the opposite side of the culture chamber wall. Using air- and gas-tight culture chambers on the RPM has the advantage of being more independent of the culture environment. However, a gas-tight culture chamber requires a culture medium that does not require CO_2_ for pH buffering, which reduces the overall cultivation period in which the culture flasks do not have to be manipulated. Gas-tight chambers in turn can cause problems when cultivating gas-producing cells, such as yeast cells.

### 4.2. Artifacts through Kinematic Rotation

In addition to a standardized cultivation method, artifacts caused by the kinematic rotation need to be considered. While the Earth's gravity vector is distributed in a way that the mean gravity converges to zero over time, the accelerations caused by the RPM's kinematics are not well controlled. In order to avoid artifacts, the rotational velocity, the sample's distance to the center of rotation, and the rotational acceleration (during velocity transitions) have to be chosen appropriately. Since there has been no systematic study on acceptable limits, scientists have to rely on their common sense. The following considerations can be used as guidelines. For explanatory reasons, we also refer here to the somewhat simpler case of clinorotation around one axis. Clinorotation and the related rotating wall vessel (RWV) bioreactor are alternative methods commonly used in many laboratories to simulate microgravity on the ground. These methods simulate microgravity by rotating samples around a horizontal axis. (Selecting the appropriate rotation velocity for suspended cells in clinostat experiments has been discussed elsewhere [17].)

**Figure 3:**
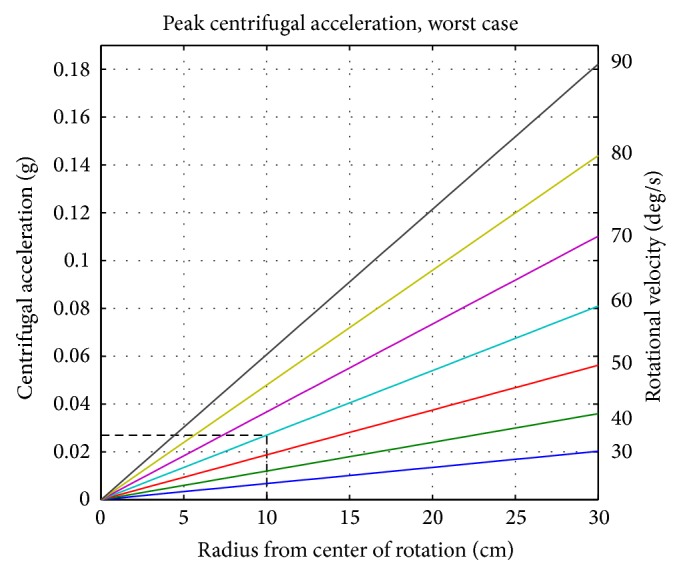
The worst-case peak centrifugal acceleration on an RPM depending on the distance to the center of rotation (*a*_pc_ ≈ 2.41 · *ω* ^2^ · *r*). For example, a moderate rotational velocity of 60 deg/s (cyan line) and a distance of 10 cm from the center of rotation (vertical dashed line) results in a peak centrifugal acceleration of approximately 0.03 g (horizontal dashed line).

**Figure 4:**
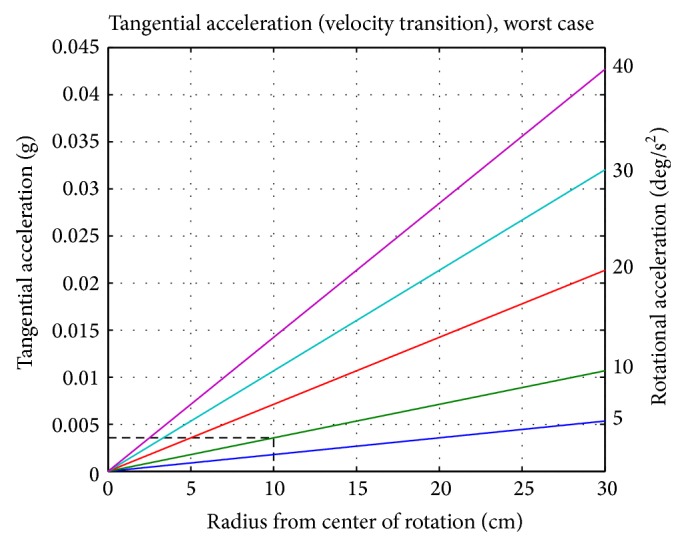
The worst-case tangential acceleration depending on the distance from the center of rotation (*a*_*t*_ = 2 · *α* · *r*). For a smooth velocity transition of, for example, 10 deg/s^2^ (green line) and 10 cm distance from the center of rotation (vertical dashed line), a tangential acceleration of approximately 0.004 g is expected (horizontal dashed line).

To minimize centrifugal acceleration, the rotational velocity and the sample's distance to the center of rotation should be set as low as the experiment allows. As mentioned earlier, the rotation shall be clearly faster than the biological processes investigated [5]. For mammalian cell experiments, many scientists have used a rotational velocity of 60 deg/s [4]. In the case of chemically activated T cells (as discussed further below), we could also create a microgravity-like environment with a rotational velocity of 40 deg/s [10]. For rotation around one axis, as in a clinostat or centrifuge, the centrifugal acceleration (in m/s^2^) is time independent and is computed by *a*_*c*_ = *ω* ^2^ · *r*, where *ω* is the rotation velocity (in rad/s) and *r* is the distance from the center of rotation (in meters). For rotations around two perpendicular axes, as is the case for RPMs, the centrifugal acceleration becomes time dependent. Thus the centrifugal acceleration depends now on the two rotation velocities, the sample's position in space and time. It is no longer trivial to make a statement on the effective centrifugal acceleration at the samples within the cultivation chamber. For the simplified case where both velocities are equal and constant, the centrifugal acceleration becomes periodically oscillating. By focusing on a worst-case scenario in terms of centrifugal acceleration, the analysis provides easy equations: in such a scenario, the peak centrifugal acceleration (in m/s^2^) can be approximated to *a*_pc_ ≈ 2.41 · *ω* ^2^ · *r* (Figure 3), where *ω* is the rotation velocity of both frames (in rad/s) and *r* is the distance from the center of rotation (in meters). As the equation indicates, all cells are ideally placed at the center of rotation. Therefore, the scientist is responsible for compactly placing the samples around the center of rotation. By using the distance to the center of rotation from the sample farthest away from this point (worst case), the largest expected centrifugal acceleration can be estimated. For a moderate velocity of typically 60 deg/s [4] and a moderate distance from the center of rotation (e.g., 10 cm), the centrifugal acceleration is in the order of 10^−2^ g. Such small forces are detectable by some specialized cells [18]. Since at any instance in time the Earth's gravity vector (which is averaged to zero over time) is present as well, the centrifugal acceleration is two orders of magnitude smaller, and we therefore consider it to be negligible. In addition, the transitions of the frames' rotational velocities introduce additional accelerations and thus should be smooth, by selecting a small rotational acceleration. For the clinostat, this tangential acceleration (in m/s^2^) is *a*_*t*_ = *α* · *r*, where *α* is the rotational acceleration (in rad/s^2^). For the RPM, the tangential acceleration becomes *a*_*t*_ = 2 · *α* · *r* in the worst case, when both frames accelerate simultaneously (Figure 4). For a smooth velocity transition of 10 deg/s^2^ and a moderate distance from the center of rotation (e.g., 10 cm), the tangential acceleration is well below 10^−2^ g.

**Figure 5:**
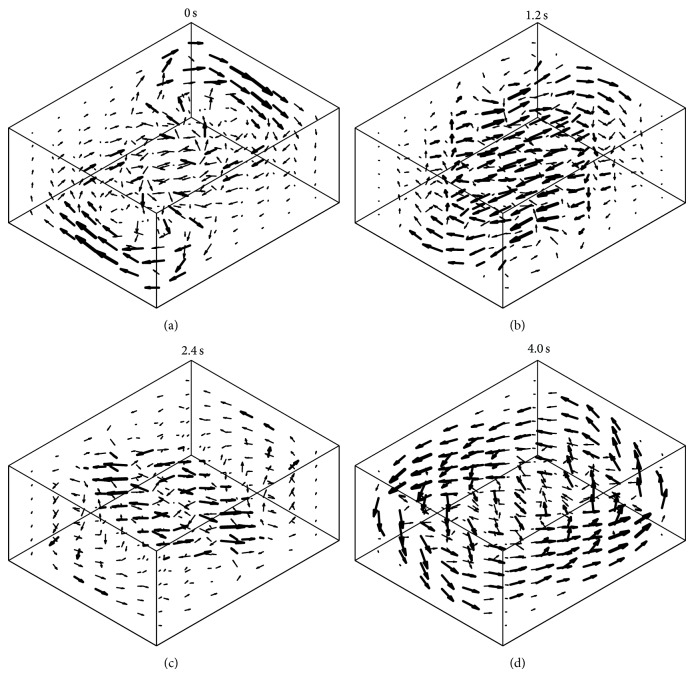
The RPM rotation introduces fluid motion in the culture flask, leading to shear forces and enhanced convection. Therefore, a moderate rotational velocity needs to be chosen, and the velocity transitions have to be smooth in order to minimize the introduction of additional mechanical stimulation of the samples. In this numerical illustration, the fluid motion is shown if both frames rotate at 60 deg/s. This results in a periodic motion of 6 seconds. The four images indicate snapshots of the velocity at 0 s (a), 1.2 s (b), 2.2 s (c), and 4 s (d).

Besides these parasitic accelerations, rotation introduces fluid motion in the culture flask, leading to shear forces and enhanced convection (Figure 5). This condition is unlike space conditions, where no convection is present. Therefore, the nutrition supply on the RPM is enhanced as compared to static or space experiments. In order to avoid additional mechanical stimulation such as shear stress, a moderate rotational velocity needs to be chosen, and the velocity transitions have to be smooth [19]. Because the behavior of fluid motion has not been fully elucidated yet, the acceptable limits for rotation velocity and acceleration are not clarified. However, the values provided above are a good starting point and have been successfully used in previous experiments [9, 10].

## 5. Experiment Reporting

As new and innovative technologies expand the range of possible experiments, it is becoming important to document the used hardware precisely. In accordance with good laboratory practice (GLP), any researcher who is using RPMs or clinostats should follow the “Bonn Criteria.” In this document it is stated that “Experimental reporting should include the properties of the culture vessel, culture media and carrier beads. These should also include dimensions and rotation speed of vessels, chemical consistency including density and viscosity of media, size, density, and porosity of beads, size, density, and porosity of cells, whether cells are motile or non-motile, density of beads with cells attached, as well as time of rotation, nature of controls, operating temperature, and gas content [20].” As described above, improper use of the RPM can introduce additional forces leading to unwanted mechanical stimulation of the sample cells. Interpreting results from such experiments could lead to wrong conclusions and could thus jeopardize a whole study.

## 6. RPM Application in Mammalian Cell Culture

### 6.1. Can the RPM Reproduce Microgravity Conditions?

Despite the long history of RPM usage, the difference between simulated and real microgravity in space shall be critically examined when interpreting experimental results. Particularly, for adhered cells, the rotation generated by the RPM could provide an unwanted source of mechanical stimuli [6]. Unfortunately, only a few researches have systematically compared experiments performed in a real microgravity environment and on an RPM. Most of these comparative studies have been done on leukocytes, for which the RPM showed good agreement with space experiments: it is well known that T lymphocytes fail to activate in microgravity after being exposed to the activator Con A [21]. This effect was reproduced numerous times on an RPM [9, 10, 22, 23]. Similarly, Villa and colleagues have shown slower proliferation of the human leukemic myelomonocytic cell line U937 exposed to simulated microgravity on the RPM [24]. The same phenomenon was previously observed on a space shuttle experiment [25]. In a study on cell mobility under microgravity with the human leukemic monocyte/macrophage cell line, the RPM predicted real microgravity results. Monocyte locomotion ability was clearly reduced in real as well as in simulated microgravity. The authors suggest that this is linked to changes in the cytoskeletal structures, since they observed reduced density of actin filaments and disruption of the *β*-tubulin architecture [26, 27]. Furthermore, peripheral blood mononuclear cells cultured for 48 hours onboard the International Space Station (ISS) showed remarkably increased apoptotic hallmarks, which could also be reproduced under simulated microgravity [28].

In recent years, two investigators directly compared the results from RPM experiments to results obtained in space conditions, performed simultaneously: in the first experiment, primary porcine chondrocytes from articular cartilage were flown for 16 days aboard the ISS. Cells exposed to microgravity showed higher collagen II/I ratio and reduced aggrecan/versican ratio at the mRNA level. In addition, cell density was significantly reduced, and the extracellular matrix straining was weaker on the ISS samples. The samples that were simultaneously exposed to simulated microgravity on an RPM generally showed results that were similar to those of the space samples but not as prominent [29]. In the second experiment, cells from the human thyroid carcinoma cell line FTC-133 were flown aboard the Shenzhou-8 spacecraft and fixed after 10 days in space. Cells exposed to spaceflight appeared to form three-dimensional tumor spheroids, while the inflight 1 g controls remained in two-dimensional monolayers. The FTC-133 cells exposed to simulated microgravity on the RPM also formed three-dimensional spheroids, even though the spheroids appeared to be smaller than those formed in space [30]. In addition, EGF and CTGF gene expression was upregulated in both real and simulated microgravity. Interestingly, EGF expression was lower and CTGF expression was higher in the RPM samples than the space samples [30]. The reason the RPM sample showed intermediate effects between the 1 g control and the space samples is not clear at this point. Since the RPM can only be used for slow processes, one possible speculation is that some of the underlying molecular processes might be too fast for RPM-simulated microgravity.

In conclusion, the RPM has been shown to mimic microgravity responses reliably for several, but not all, experimental conditions. Particularly, for leukocytes, several effects seen in space were reproduced on the RPM. Particular studies designed to investigate differences in cellular responses between space samples and samples exposed to simulated microgravity elucidated an underestimation or overestimation of simulated versus real microgravity. Overall, the RPM generally seems to underestimate the spaceflight effects. Therefore, results from RPM experiments need to be interpreted with caution and, if possible, more directly compared to experiments under real microgravity in order to fully assess their capability to support gravitational biology studies.

### 6.2. Novel Applications of the RPM

The exact mechanisms by which mechanical stimuli initiate cellular modifications have still not been fully elucidated [31]. This is the motivation of mechanobiologists to expose cells to various mechanical stimuli such as distinct patterns of shear flow, tensile stretch, or mechanical compression at various parametric combinations of magnitude, duration, or frequency [31]. The RPM can be regarded as an additional mechanical device for reducing the long-term effects of the mechanical force of gravity. Due to the constant reorientation of samples on the RPM, gravity-dependent intracellular responses will not be triggered anymore. Thus one can say that the RPM generates a state of a mechanically unloaded environment in which the longer-lasting impact of gravity can be studied.

**Figure 6:**
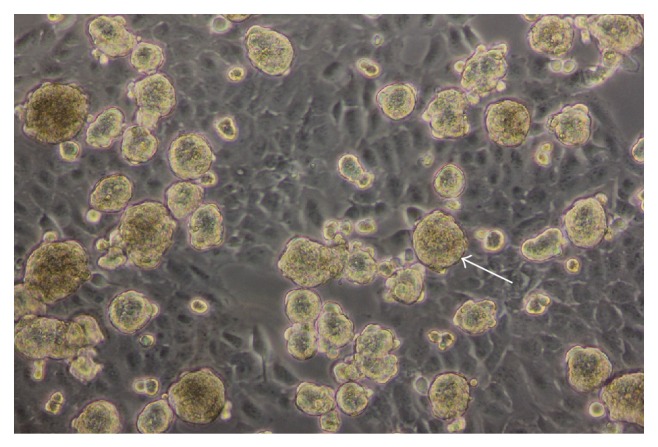
Thyrocytes cultured for seven days on the RPM organized to spheroid structures (arrow).

Monolayer (two-dimensional) cell cultures have been successfully used for many decades, allowing a better understanding of many cellular and molecular processes. They actually represent an important source of information prior to animal experimentation. Despite numerous advantages, the monolayer model cannot simulate organs or tissues realistically. Therefore, three-dimensional cell culturing has emerged over the last decades as an alternative to mimic better tissue-like organization with the idea of closing the gap of uncertainty between tissue-like and monolayer cell culture. The RPM in that context appears as an alternative approach to generating a three-dimensional culture [32]. The random repositioning of the cells around the gravity vector over time allows constant redistribution of gravity forces, which thus leads to the formation of cell aggregates that can form microspheroids (Figure 6) [32–34]. Spheroids organized as multilayers are closer to* in vivo* tissue situation than monolayer cells [32]. Such samples are therefore more accurate as a model integrating the three-dimensional real surroundings of a cell in an* in vivo* tissue. Thus, spheroid structures open a new field of applications, such as test systems for drug therapies or diagnosis [35]. The spheroid structure is actually a good model to screen for penetration characteristics of drugs or antibodies through tissue.

## 7. Conclusion

Several RPMs have evolved during the past years that feature different designs, functions, and motion patterns. They all have reliably proven to simulate microgravity conditions. Developments to RPM hardware and software have expanded the experimental possibilities substantially. The successful operation of digital holographic microscopy (DHM) on the RPM and the implementation of partial gravity algorithms have opened new fields in gravitational research, particularly in mechanobiology.

In order to obtain reliable and comparable data, the appropriate use of the RPM and application of standardized cultivation methods are of central importance. The RPM has been established as a reliable tool supporting ground-based microgravity studies. Effects seen in real microgravity were reproduced with good agreement on RPMs. Some RPM studies, however, also showed cellular effects that were between those of the real microgravity and 1 g ground control results. The RPM is furthermore an ideal tool for preliminary microgravity tests, screening studies in which simulated microgravity effects are checked on various organisms and hardware testing. Particularly, for suggesting live science experiments for the conduction under real microgravity in space, the presentation of preliminary data showing modifications under simulated microgravity is becoming very important. Advances in RPM engineering and live science qualify the RPM as an interesting tool for novel applications, such as three-dimensional cell culturing as well as tissue engineering.
